# Efficacy of Hemocoagulase as a Topical Hemostatic Agent After Dental Extractions: A Systematic Review

**DOI:** 10.7759/cureus.2398

**Published:** 2018-03-30

**Authors:** Gauri Gupta, Muthusekhar M.R., Santhosh P Kumar

**Affiliations:** 1 Oral and Maxillofacial Surgery, Saveetha Dental College, Saveetha University, Chennai

**Keywords:** hemostasis, hemocoagulase, bleeding, wound healing, extraction, oral surgery, local hemostatic agent, saline pressure packs

## Abstract

Extraction is one of the more common oral surgical procedures carried out in routine dental practice, and postextraction bleeding is a recognized, frequently encountered complication. It causes distress, agony, and discomfort to the patient. The aim of this systematic review was to analyze the existing literature and determine the efficacy of topical hemocoagulase as a hemostatic agent and its ability to reduce postoperative complications in comparison to routine saline pressure pack after the extraction of teeth. Information was collected from an electronic database (PubMed), and a manual search was also done in Oral Surgery, Oral Medicine, Oral Pathology, Oral Radiology; International Journal of Oral and Maxillofacial Surgery; and the British Journal of Oral and Maxillofacial Surgery. Only those articles which met the inclusion criteria were selected. All studies and articles that compared topical hemocoagulase with saline pressure pack in patients requiring extraction of teeth were selected for review. Literature abstracts and full-text articles were analysed in this review. A total of four articles were included in this systematic review. All were randomized clinical trials that evaluated the clinical outcomes of topical hemocoagulase compared with saline pressure packs in extraction socket sites. A significant difference was present between the hemocoagulase group and control group (saline pressure pack) in relation to bleeding stoppage time, pain, swelling, wound healing, and other postoperative complications. Topical hemocoagulase is significantly effective in reducing bleeding, pain, and swelling after extraction of tooth when compared to saline pressure packs. It also acts as a promoter of wound healing.

## Introduction and background

Bleeding during oral surgical procedures can cause distress, agony, and discomfort to the patient. It also distracts the oral surgeon from operating, leading to frustration and time consumption. Tooth removal or extraction is one of the more common invasive oral surgical procedures carried out in routine dental practice, and postextraction bleeding is a recognized, frequently encountered complication in dental practice [1]. Postextraction bleeding is defined as “evidence of bleeding beyond the pressure pack” [2]. There is a wide array of techniques suggested for the management of postextraction bleeding, both in healthy and medically compromised patients [3-5]. Interventions for managing postextraction bleeding can be broadly categorized into local and systemic interventions [6]. Local intervention includes common saline pressure pack, sutures, acrylic surgical splints, and local hemostatic agents [7]. Local hemostatic agents used in oral surgery are classified into passive and active hemostatic agents. They include oxidized cellulose, resorbable gelatin sponge, collagen sponge, polysaccharide hemospheres, thrombin, cyanoacrylate glue, fibrin glue adhesive, local antifibrinolytic solutions such as tranexamic acid mouthwash, bone wax, ostene, calcium alginate, chitosan-based dressings, polysaccharide-based hemostats, tannic acid, and hemocoagulase [8-10]. If necessary, systemic hemostatic agents can be used for achieving hemostasis.

Hemocoagulase, a fractional isolate of poisonous Bothrops jararaca, is an enzyme complex. It accelerates the formation of fibrin monomers and hastens fibrin clot formation [11]. The application of hemocoagulase after extraction produces reduced bleeding stoppage time, postoperative pain, and swelling after the extraction of teeth [12]. Hemocoagulase is contraindicated in venous and arterial thrombosis, and in patients with a tendency for intravascular coagulation. It can be applied topically or administered through intramuscular or intravenous routes depending upon the condition to be treated.

A systematic review based on the highest clinical evidence will indicate the benefits of hemocoagulase in the management of postoperative bleeding after dental extractions [3]. Hence, the aim of this systematic review was to compare the efficacy of hemocoagulase as a topical hemostatic agent with routine saline pressure pack after tooth extraction.

## Review

Structured question

Is local application of hemocoagulase a simple and effective way of reducing postoperative complications as compared to saline pressure pack after extraction of teeth?

Population, intervention, comparison, and outcome analysis

Our population, intervention, comparison, and outcome (PICO) analysis was as follows:

P: Patients requiring more than one extraction such as full mouth extraction for dentures or extractions for orthodontic treatment

I: Topical hemocoagulase solution

C: Saline pressure pack

O: Postoperative bleeding, pain, and swelling

Inclusion criteria

Clinical trials, comparison studies, and studies only in English were included in this review.

Types of participants/samples

Patients requiring more than one extraction (orthodontic intervention necessitating bilateral removal, full mouth extraction for dentures, bilateral impacted lower third molar teeth)

Types of intervention

1. Topical hemocoagulase

2. Saline pressure pack

Outcome measures

Postoperative bleeding, pain, and swelling, wound healing

Exclusion criteria

Animal studies and literature in other languages were excluded from the review process.

Literature search

Information was collected from an electronic database (PubMed). A search was also done in Oral Surgery, Oral Medicine, Oral Pathology, Oral Radiology; International Journal of Oral and Maxillofacial Surgery; British Journal of Oral and Maxillofacial Surgery to date.

Search methodology

The search methodology was a combination of Medical Subject Headings terms and suitable keywords based on PICO formulated for the review. The terms used were dental extractions, hemocoagulase, botropase, saline pressure packs, hemostasis, postoperative pain, and swelling.

Selection of studies

The review process consisted of two phases. In the first phase, the title and abstracts of the articles obtained through the PubMed search were examined for relevance. The full text of relevant articles was obtained and accessed. In the second phase, relevant articles were isolated based on inclusion and exclusion criteria for further data extraction and statistical analysis. All studies that compared topical hemocoagulase with saline pressure pack in patients requiring extraction of teeth were selected for review.

Data extraction and quality evaluation

Data extraction for general characteristics of all included studies and variables of outcomes were done. All articles identified as suitable were read in their entirety, considered for inclusion, and summarized based on general characteristics such as sample size, study groups, treatment, methods of evaluation, results, statistical analysis, and inference.

Variables of interest were postoperative bleeding stoppage time, where the time was measured from application of the material into the socket or surgical site up to the complete stoppage of bleeding by using a stopwatch, pain, and swelling. Levels of evidence of included studies and risk of bias were done for all the studies. Outcomes assessment and risk of bias for the included studies were assessed based on the consort criteria. The study was assessed to have a “high risk” of bias if it did not record a “yes” in three or more of the four main categories; “moderate” if two out of four categories did not record a “yes”; and “low” if randomization, assessor blinding, and completeness of follow-up was considered adequate.

**Figure 1:**
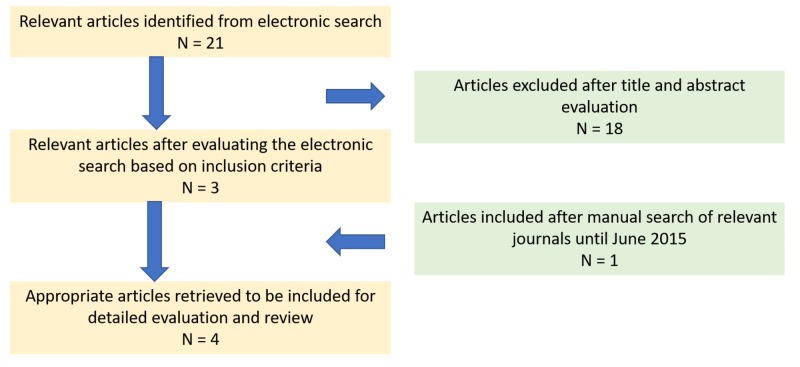
PRISMA flowchart for selection of studies. Abbreviations: PRISMA, Preferred Reporting Items

The electronic database search identified 21 articles based on the query. After reading titles and abstracts, 18 studies were excluded as they were irrelevant for the review. After examination of full texts, the remaining three studies were included in the review. After a manual search of articles, one relevant article was added to the review process (Figure 1).

Finally, four articles were included for review in the present study, and their characteristics are described in Table 1 and Table 2.

**Table 1:** Characteristics of included studies: Methodologies

S. NO	AUTHOR AND JOURNAL	SAMPLE SIZE	STUDY LOCATION	TREATMENT/ INTERVENTION	METHODS OF EVALUATION
1	Aslam et al. The Journal of Contemporary Dental Practice. 2013 [20]	20 (40 surgical sites) [20]	Yenepoya University India [20]	Forty extractions for orthodontic reasons were done in 20 patients bilaterally. Two groups of 20 samples each: Group A (Site A): 1 cc of hemocoagulase was locally applied in the socket following extraction. Group B (Site B) (control group): Placebo, no hemocoagulase was applied after extraction of tooth [20]	Bleeding stoppage time was recorded using a stopwatch to record the time from placement of solution up to complete formation of clot. Pain at six hours: Postoperatively, one information chart in the form of a visual analogue scale was given to each patient for evaluating postoperative pain [20]
2	Majumder et al., International Journal of Clinical Medicine. 2014 [21]	25 (50 surgical sites) – group 1 50 (100 surgical sites) – group 2 [21]	SGT University India	Fifty simple extractions (for complete dentures or for orthodontic reasons) were done. Fifty bilateral impactions (100 extractions) were done. Site A: Hemocoagulase was used topically by local irrigation to control hemorrhage. Site B (control): No drug was used to control the hemorrhage [21]	Bleeding stoppage time was recorded using a stopwatch to record the time from placement of solution up to complete formation of clot. Pain at six hours: Postoperatively, one information chart in the form of a visual analogue scale was given to each patient for evaluating postoperative pain. Swelling reference points: Lateral corner of the eye to angle of the mandible and tragus of the ear to the corner of the mouth. Postoperatively, study parameters were recorded after one hour, two hours, three hours, six hours, and nine hours [21]
3	Joshi et al., Annals of Maxillofacial Surgery. 2014 [22]	200 (400 surgical sites) [22]	Bharati Vidyapeeth University India [22]	A total of 200 patients who underwent symmetrical bilateral orthodontic extractions were analyzed for bleeding stoppage time. After extraction of teeth on one side, hemocoagulase (1U) was applied, and on the other side, pompom impregnated with placebo (1 mL normal saline [NS]) was applied [22]	Mean bleeding stoppage time was evaluated using a stopwatch [22]
4	Solanki et al., International Journal Research in Medical Sciences. 2015 [23]	100 (200 surgical sites) [23]	PDU University India [23]	Extraction of grossly decayed or non-restorable teeth, or due to severe periodontitis in 100 patients (200 sites) were included in the study. At the first site after extraction, the extraction socket was filled with hemocoagulase solution (test site). After 24 to 48 hours, extraction procedure was carried out on another site (control site) and saline pressure pack was placed [23]	Bleeding stoppage time Test site: Measurement of time from application of hemocoagulase solution into the socket until the complete stoppage of bleeding was calculated using a stopwatch. Control site: Saline pressure pack was given to achieve hemostasis at this site and the time taken for hemostasis was calculated. Swelling reference points: Lateral corner of the eye to the angle of the mandible and tragus of the ear to the corner of the mouth. Swelling was measured on the first, second, third, fourth, and fifth days [23]

**Table 2:** Characteristics of included studies: Findings

S. NO	AUTHOR AND JOURNAL	STUDY GROUPS	STATISTICAL ANALYSIS	RESULTS			INFERENCE
1	Aslam et al., The Journal of Contemporary Dental Practice. 2014 [20]	1. Hemocoagulase solution 2. Placebo [20]	‘t’ test	1. Bleeding stoppage time: Test site (hemocoagulase): 1.3735 ± 0.5218 Control site (saline pack): 2.3275 ± 0.8480 2. Pain at 6 hours: Test site (hemocoagulase): 20.0 ± 32.163 Control site (saline pack): 55.5 ± 37.032 [20]			Hemocoagulase group achieved faster hemostasis when compared to control group. The results were highly significant. Amount of pain on site A (test) was found to be less as compared to site B (control). The result was statistically significant [20].
2	Majumder et al., International Journal of Clinical Medicine. 2014 [21]	1. Hemocoagulase solution (Test site) 2. No drug (Control site) [21]	The student t-test for equality of means	1. Bleeding stoppage time: Test site (hemocoagulase): 1.3532 ± 0.432 Control site (saline pack): 2.2532 ± 0.859 2. Pain at six hours: Test site (hemocoagulase): 16.6 ± 1.83 Control site (saline pack): 50.6 ± 6.12 3. Swelling: Test site (hemocoagulase): One hour: 26.23 ± 6.1 Two hours: 14.5 ± 1.8 Three hours: 1.023 ± .5 Six hours: 0 Nine hours: 0 Control site (saline pack): One hour: 40.6 ± 4.8 Two hours: 32.6 ± 4.6 Three hours: 18.1 ± 2.1 Four hours: 0 Five hours: 0 [21]			Faster hemostasis was achieved at hemocoagulase site (p = 3.95). The result was statistically significant. Amount of pain on site A (test) was found to be less as compared to site B (control). The result was statistically significant (p = 0.01). Hemocoagulase site: 13 patients (26%) had swelling after one hour and reduced to seven patients (14%) by two hours. At control site, 20 patients (40%) had swelling after one hour and reduced to 16 patients (32%) by three hours (p = 0.02). The result was statistically significant [21].
3	Joshi et al., Annals of Maxillofacial Surgery. 2014 [22]	1. Hemocoagulase (test site) 2. Standardized pompom impregnated with placebo (1 mL) normal saline (control site) [22]	Unpaired ‘t’ test	Bleeding stoppage time: Test site (hemocoagulase): 1.6732 ±0.5319 Control site (saline pack): 3.0316 ± 1.445 [22]			The analysis revealed p < 0.001 for bleeding stoppage time in the study group, which is highly significant [22].
4	Solanki et al., International Journal of Research in Medical Sciences. 2015 [23]	1. Hemocoagulase (test site) 2. Saline pressure pack (control site) [23]	Unpaired ‘t’ test	1. Bleeding stoppage time: Test site (hemocoagulase): 1.5231 + 1.445 Control site (saline pack): 3.311 + 1.316			Faster hemostasis was achieved on hemocoagulase site. In terms of swelling, there was not much differences on both the sites on the second and third postoperative days [23].
				2. Swelling:			
				Time	Test site	Control site	
				First day	NA	40.8 ± 4.7	
				Second day	50.16 ± 2.13	60.12 ± 3.126	
				Third day	30.061 ± 1.23	36.126 ± 2.16	
				Fourth day	10.012 ± 0.31	14.32 ± 1.675	
				Fifth day	NA	4.069 ±0.653	

**Figure 2:**
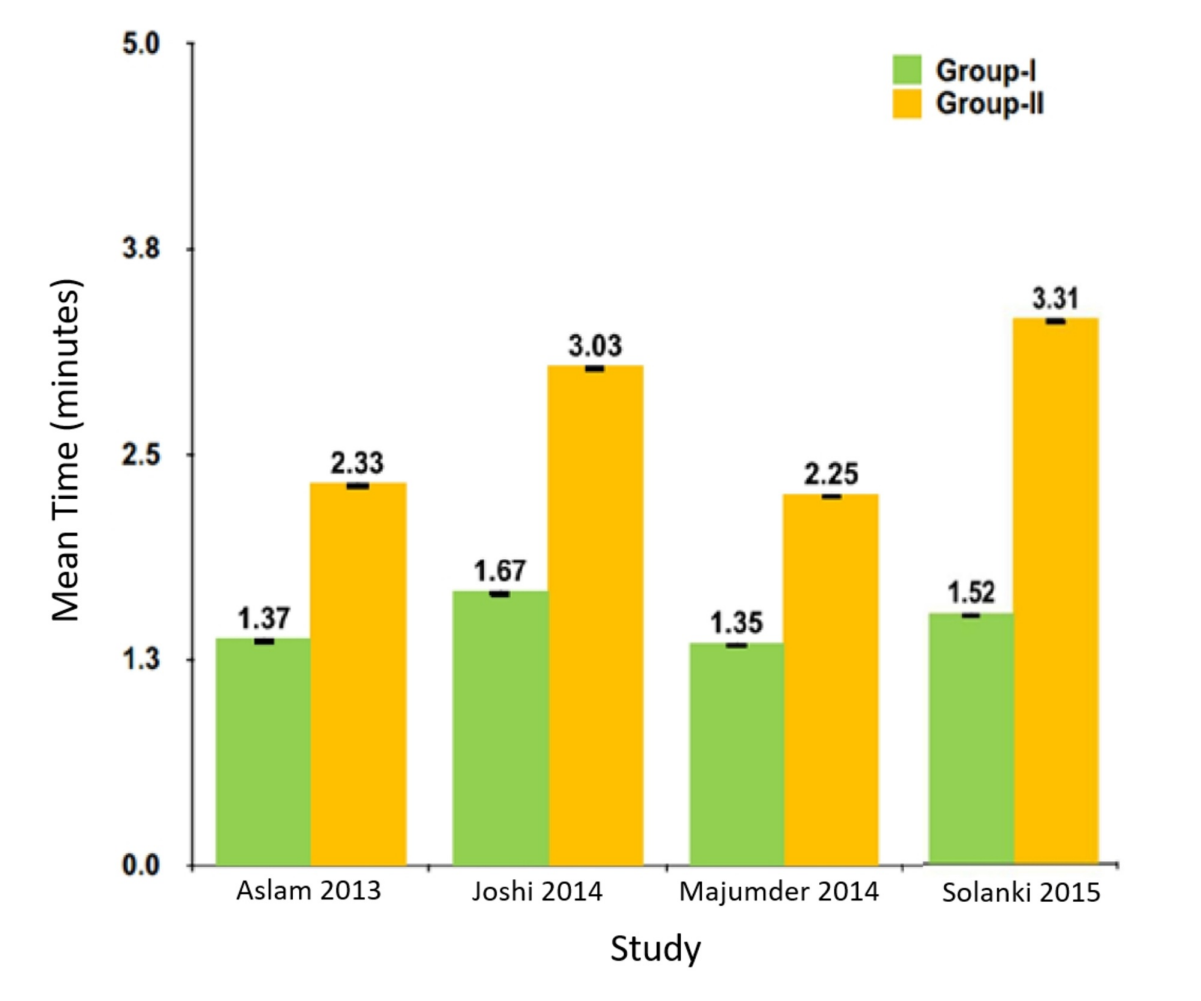
Mean bleeding stoppage time in included studies

In all the studies, mean bleeding stoppage time was less in the hemocoagulase group when compared to the control group. The difference was statistically significant (Figure 2).

**Figure 3:**
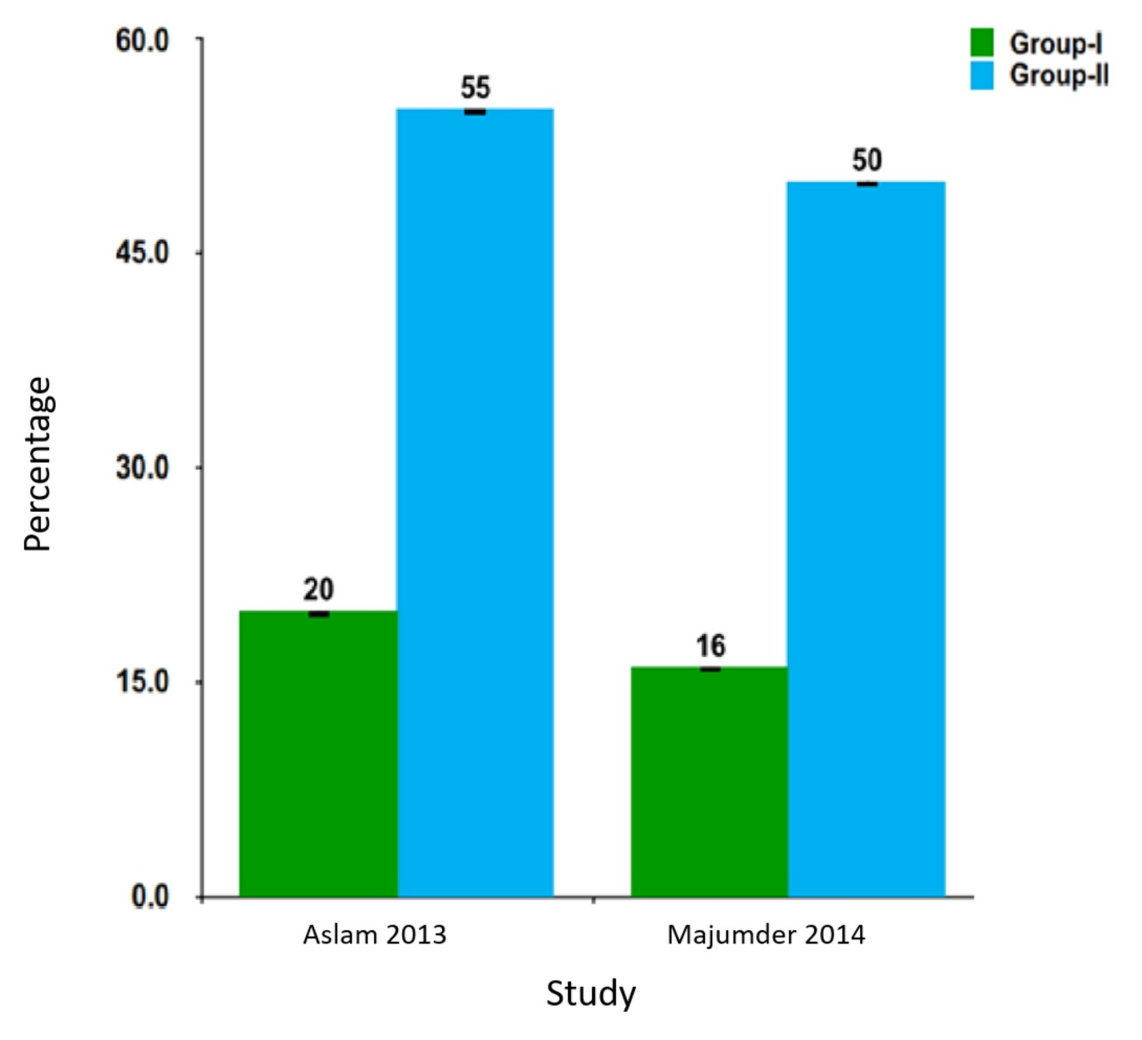
Pain scores in included studies

Pain scores at the sixth hour were significantly less in the hemocoagulase groups (Figure 3).

**Figure 4:**
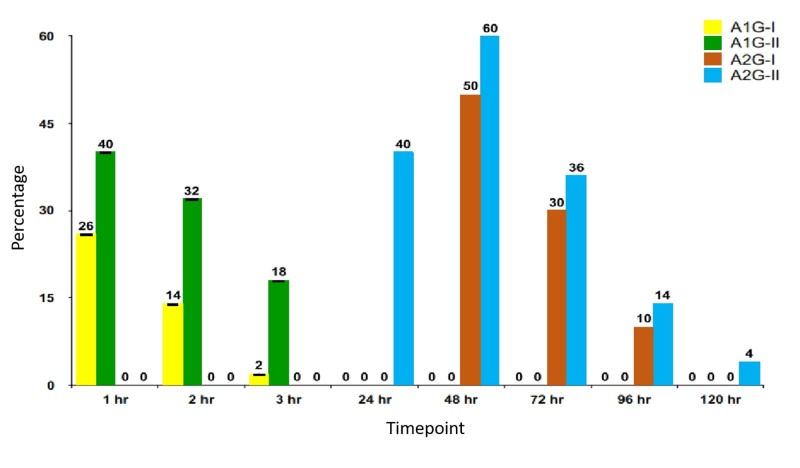
Prevalence of swelling in included studies

Swelling was more in placebo or control (saline pressure pack) sites than in hemocoagulase sites (Figure 4).

Levels of evidence

The levels of evidence for the reviewed studies are based on the Oxford Centre for Evidence-Based Medicine (Table 3).

**Table 3:** Levels of evidence in included studies

S. No.	Author and Year	Study Design	Levels of Evidence
1	Aslam et al., 2013 [20]	Case control	3b
2	Majumdar et al., 2014 [21]	Prospective study	2b
3	Joshi et al., 2014 [22]	Randomized controlled trial	1
4	Solanki et al., 2015 [23]	Randomized controlled trial	1

Risk of bias

The four main methodological studies were assessed for quality, and the included studies showed low to moderate risk of bias (Table 4).

**Table 4:** Risk of bias in included studies

Study	Randomization	Allocation Concealed	Assessor Blinding	Dropouts Described	Risk of Bias
Aslam et al., 2013 [20]	No	Yes	Yes	None	Moderate
Majumder et al., 2014 [21]	No	Yes	Yes	None	Moderate
Joshi et al., 2014 [22]	Yes	Yes	Yes	None	Low
Solanki, et al., 2015 [23]	Yes	Yes	Yes	None	Low

Benefits of hemocoagulase as a topical hemostatic agent

Extraction of teeth is a common procedure in oral and maxillofacial surgery. Because the region of surgery is mostly composed of loose connective tissue that contains blood and lymph vessels, a series of functional and structural alteration is expected after extraction, mostly expressed as pain, prolongation of bleeding stoppage time, and swelling [13-14]. Hemocoagulase is a fractional isolate of poisonous Bothrops jararaca or Bothrops atrox and is an enzyme complex, with coagulative and anti-hemorrhagic properties [15-16]. Being a topical form, it also acts fast and is atoxic. Hemocoagulase has enzymatic actions similar to thromboplastin and thrombin, and thereby promotes rapid blood coagulation and wound healing [10].

Many studies have evaluated the healing mechanism of the extraction socket wound and the physiological changes that occur at the cellular level immediately after extraction [17-19]. There have been studies that have evaluated the role of hemocoagulase in reducing postoperative bleeding stoppage time in patients requiring extraction of teeth. Other parameters such as postoperative pain, swelling, wound healing, and infection rates were also evaluated.

Aslam et al. [20] conducted a study on 20 subjects to evaluate the efficacy of local application of hemocoagulase solution as compared to a placebo in wound healing following dental extraction. Bleeding stoppage time was recorded using a stopwatch to record the time from placement of solution until the complete formation of the clot. The differences between the hemocoagulase site and control site were significant. Pain was also evaluated at the sixth hour, and the amount of pain in the hemocoagulase group was less compared to the placebo group, and the results were statistically significant. The authors concluded that the topical hemocoagulase solution could be used as a hemostatic agent and promoter of wound healing in oral surgery.

Majumder et al. [21] evaluated the efficacy of topical hemocoagulase on 50 surgical sites in 25 patients who underwent simple extractions. Parameters such as bleeding stoppage time, pain, and swelling were measured. Time taken for hemostasis was calculated using a stopwatch. Facial swelling was recorded using measurements between the lateral corner of the eye to the angle of mandible and tragus of the ear to the corner of the mouth. Postoperatively, one information chart in the form of a visual analogue scale was given to each patient to evaluate pain. A significant difference was found in bleeding stoppage time, postoperative pain, and swelling between the test (hemocoagulase) and control (no drug) group. The authors concluded that use of hemocoagulase solution after extractions not only provides faster hemostasis but also enhances healing by rapid formation of healthy tissue with less chance of infection. Based on these results, it was suggested that further studies were required to prove the use of hemocoagulase in hemophilic patients.

Joshi et al. [22] compared bleeding stoppage time between the test (hemocoagulase) and the control (pompom impregnated with placebo [1 mL normal saline]) groups after symmetrical bilateral orthodontic extractions. A statistically significant difference was present between the test and the control groups. The authors concluded that hemocoagulase holds good prospects in managing postextraction bleeding in cardiac patients on aspirin without stopping aspirin before extraction. Its topical use provides faster hemostasis in patients undergoing dental extraction without any systemic or local adverse effect.

In a study by Solanki et al. [23], the researchers compared bleeding stoppage time, pain at the sixth hour, and swelling between the test (hemocoagulase) and control (saline pressure pack) groups after undergoing extractions. There were statistically significant differences between these groups on evaluation. The authors concluded that application of hemocoagulase to extraction socket will achieve faster hemostasis, reduce pain and swelling, and help in wound healing by rapid formation of healthy tissue. It also has the advantage of reducing the amount of infection in the extraction sockets.

Shenoy et al. [24] evaluated the effects of topical hemocoagulase on intra-oral extraction sockets as well as the impact on the healing process. In this study design, topical hemocoagulase was placed in one site while the other site did not receive anything. Clinical evaluation was done on the following postoperative days seven, 14, and 21 for all patients. Biopsies were done on a random basis either on day seven or 14 for cases as well as controls. A clinical and histopathological score was developed and wound healing was assessed. The clinical score did not show any statistical significance. However, the histopathology score had an increased incidence of osteoid formation in the hemocoagulase group on day 14. The authors concluded that the application of hemocoagulase might improve and accelerate the process of wound healing in an extraction socket. This study was not included in the systematic review, as no comparable data with the previous studies were present.

From these studies, a single postoperative hemocoagulase application has been shown to reduce postoperative pain and swelling after dental extractions. Rapid hemostasis with the uneventful healing of extraction wounds with the use of hemocoagulase was observed in all these studies, and they emphasize that hemocoagulase plays an important role as a promoter of wound healing [20-24].

## Conclusions

Based on the clinical evidence, topical hemocoagulase is an effective hemostatic agent after dental extractions. It also reduces pain and swelling and promotes wound healing by rapid formation of healthy tissue. Further large-scale clinical trials are necessary to explore additional applications of topical hemocoagulase solution in the field of oral and maxillofacial surgery.
